# GSIDroid: A Suspicious Subgraph-Driven and Interpretable Android Malware Detection System

**DOI:** 10.3390/s25134116

**Published:** 2025-07-01

**Authors:** Hong Huang, Weitao Huang, Feng Jiang

**Affiliations:** 1 School of Computer Science and Engineering, Sichuan University of Science & Engineering, Zigong 643000, China; 2 School of Artificial Intelligence, Nanjing University of Information Science and Technology, Nanjing 210044, China

**Keywords:** Android malware detection, semantic information, graph convolutional network, explainable machine learning

## Abstract

In recent years, the growing threat of Android malware has caused significant economic losses and posed serious risks to user security and privacy. Machine learning-based detection approaches have improved the accuracy of malware identification, thereby providing more effective protection for Android users. However, graph-based detection methods rely on whole-graph computations instead of subgraph-level analyses, and they often ignore the semantic information of individual nodes. Moreover, limited attention has been paid to the interpretability of these models, hindering a deeper understanding of malicious behaviors and restricting their utility in supporting cybersecurity professionals for further in-depth research. To address these challenges, we propose GSIDroid, a novel subgraph-driven and interpretable Android malware detection framework designed to enhance detection performance, reduce computational overhead, protect user security, and assist security experts in rigorous malware analysis. GSIDroid focuses on extracting suspicious subgraphs, integrating deep and shallow-semantic features with permission information, and incorporating both global and local interpretability modules to ensure transparent, trustworthy, and analyzable detection results. Experiments conducted on 14,520 samples demonstrate that GSIDroid achieves an F1 score of 97.14%, and its interpretability module successfully identifies critical nodes and permission features that influence detection decisions, thereby enhancing practical deployment and supporting further security research.

## 1. Introduction

As of 2024, there are approximately 6.8 billion smartphone users worldwide. When including all mobile devices—such as tablets, feature phones, and wearables—the number of connected mobile devices exceeds 16 billion. This figure reflects not only the prevalence of individuals owning multiple devices, but also the growing integration of the Internet of Things (IoT) into everyday life [1]. With the continuous advancement of information technology, cyberspace is evolving at an unprecedented pace. However, this rapid development has also opened new avenues for cybercriminals. In particular, the proliferation of Android-targeted malware has become a growing concern. According to recent reports, the proportion of mobile applications subjected to attacks increased from 65% in February 2024 to 82.7% by January 2025, indicating that mobile platforms—including both iOS and Android—are facing unprecedented security threats [2]. These malware variants employ a wide range of attack strategies, significantly increasing the risks of financial loss and personal privacy breaches for users. For instance, certain malware is capable of stealing sensitive user information, executing unauthorized financial transactions, or silently installing additional malicious applications without user awareness.

Given the increasingly severe security landscape, advancing malware detection technologies have become an urgent priority. Existing detection methods are generally classified into two major categories, namely, traditional detection approaches and machine learning-based approaches [3,4]. Traditional detection techniques typically extract configuration files or source code and identify vulnerabilities or malicious behaviors through predefined rules or signature matching. These methods rely heavily on domain experts to craft detailed rule sets and maintain large-scale signature databases. Although such techniques were instrumental during the early stages of malware defense, they exhibit significant inherent limitations. First, traditional methods are highly dependent on the continuous updating of signature databases, as new malware variants emerge rapidly and render existing signatures obsolete. Second, Android applications can easily evade signature-based mechanisms through straightforward code obfuscation techniques, substantially undermining detection effectiveness [4,5]. Moreover, the process of writing and maintaining rule sets is both time-consuming and resource-intensive, and it remains difficult to comprehensively address all potential threats, especially novel or previously unseen attack patterns.

By leveraging machine learning techniques, it is possible to overcome many of the limitations associated with traditional malware detection approaches. Deep learning, a prominent subfield of machine learning, has achieved significant breakthroughs in areas such as computer vision (CV) and natural language processing (NLP) [6,7]. Building on these successes, recent research has increasingly explored the application of deep learning methods to malware detection, with the goal of automatically learning from existing behavioral patterns and identifying newly emerging malware threats to mitigate the rapidly escalating risks in the mobile ecosystem. Within deep learning-based frameworks, feature extraction plays a pivotal role, as the quality and relevance of extracted features directly determine the overall detection performance of the model.

Currently, three primary feature extraction approaches are employed for Android malware detection, namely, static analysis, dynamic analysis, and hybrid analysis [8]. Static analysis aims to examine an application’s code without executing it, typically by parsing the APK file structure and extracting features from bytecode or binary code. This approach offers high efficiency, enabling the rapid processing of large volumes of applications. İbrahim et al. [9] utilized static analysis to extract a variety of features from Android applications for classification purposes. Kiraz et al. [10] extracted static features, converted them into embedding vectors using a BERT-based model, generated image representations, and subsequently performed classification with convolutional neural networks (CNNs). Niu et al. [11] compressed large heterogeneous APK-API relational graphs into homogeneous APK graphs and extracted reachability relationships, thereby improving both detection efficiency and classification accuracy. Nevertheless, static analysis remains vulnerable to code obfuscation and packing techniques, which can severely hinder its ability to detect sophisticated or heavily disguised malware.

Dynamic analysis involves executing applications within a controlled environment to observe their behavior and the resulting system changes, such as memory usage, battery consumption, network traffic, and API call sequences. Cui et al. [12] proposed DroidHook, a novel dynamic analysis sandbox framework for Android malware. NT-GNN [13] extracts network traffic graphs and leverages a graph neural network (GNN) architecture for malware detection. Although dynamic analysis provides a more accurate reflection of an application’s real-world behavior, it is typically time-consuming and struggles to achieve complete behavioral coverage, especially for malicious logic that is triggered only under specific conditions.

Hybrid analysis combines static and dynamic analysis techniques, leveraging both statically extracted features and dynamically captured behaviors to identify malware. HGDetector [3] enhances malware detection accuracy and efficiency by employing a multi-feature fusion approach that integrates network traffic data and function call graphs (FCG). While hybrid analysis can effectively capitalize on the strengths of both static and dynamic methods, thereby improving detection accuracy and reliability, it also faces notable challenges. Specifically, the process can be excessively complex and resource-intensive, which may limit its scalability and practicality in large-scale deployment scenarios.

Moreover, most existing malware classifiers are designed as “black-box” models, lacking transparency and interpretability, and thus fail to provide meaningful insights for security experts to conduct further analysis. Although some studies have attempted to reveal the decision-making processes of models through interpretability techniques [14,15,16], these approaches often fall short in deeply exploring the underlying control flow logic and struggle to accurately identify specific malicious code fragments within malware samples.

Considering the strengths and weaknesses of various analysis methods, we adopt static analysis as the primary foundation of our approach. Static analysis is well-suited for large-scale malware detection tasks due to its efficiency and relatively low resource consumption. However, existing static analysis-based graph construction techniques often rely on whole-graph computation, which imposes significant computational overhead and demands high processing power. More importantly, these methods typically emphasize structural features while neglecting the underlying semantic information embedded within functions, thereby limiting both the robustness and accuracy of the detection models. To address these limitations, our goals are as follows: (1) to enhance the semantic representation of malware by integrating both deep and shallow-semantic features, and (2) to improve the interpretability of detection results to support in-depth security analysis. To this end, we propose GSIDroid, a novel subgraph-driven and interpretable Android malware detection system designed to capture semantically suspicious substructures while providing both global and local explanations of the classification outcomes.

Our main contributions are summarized as follows:

1. Subgraph-driven computation for efficiency and relevance: Instead of performing computation over the entire graph, we focus on extracting suspicious subgraphs centered around sensitive APIs. This approach significantly reduces computational overhead and improves processing efficiency, while preserving the most critical structural and behavioral information within the graph.
2. A novel architecture incorporating semantic and permission features: We design a new network architecture that integrates semantic information into the graph representation. Beyond structural features, our model fuses both deep and shallow-semantic representations along with permission features through a dedicated feature integration module. This enables more precise identification of subgraphs containing malicious behaviors.
3. Dual-level interpretability for enhanced transparency: To improve the transparency and interpretability of malware detection results, we introduce a dual-level explanation module: a global explanation based on permission usage statistics, and a local explanation using node-level contribution scores. These explanations help analysts better understand and investigate the model’s decision-making process.

The remainder of this paper is organized as follows. Section 2 reviews related work in the field of Android malware detection. Section 3 introduces the proposed GSIDroid framework and provides detailed descriptions of its core components. Section 4 outlines the experimental setup. Section 5 presents comprehensive experiments conducted on benchmark datasets and evaluates the performance of the proposed model as well as the effectiveness of each module. Section 6 demonstrates the interpretability of GSIDroid through both global and local explanation mechanisms. Finally, Section 7 concludes the paper and discusses potential directions for future research.

## 2. Related Work

In recent years, malware detection has witnessed a significant paradigm shift from pattern-driven approaches to data-driven methodologies [17]. This shift highlights the growing preference for deep learning techniques over traditional signature-based detection methods and handcrafted feature engineering. By enabling automated feature extraction, deep learning models substantially reduce reliance on expert knowledge and simplify the feature design process. One common line of research involves converting the DEX files of Android applications into grayscale images—such as through bytecode visualization—to leverage computer vision techniques for capturing texture patterns and spatial structures indicative of malicious behavior [18,19,20,21]. In parallel, other studies have focused on the semantic analysis of DEX files by examining opcode sequences, aiming to extract higher-level behavioral patterns [22,23]. It is also important to note that malicious applications often execute harmful behaviors by accessing sensitive APIs or requesting high-risk permissions. Accordingly, a considerable body of work [24,25,26,27] has explored the extraction of API call traces and permission usage as discriminative features for effective malware identification.

The effectiveness of the aforementioned detection methods has been significantly driven by the rapid advancement of technologies such as convolutional neural networks (CNNs), recurrent neural networks (RNNs), and natural language processing (NLP). In recent years, graph neural networks (GNNs) have emerged as a promising paradigm, owing to their capacity to model relational and structural information inherent in graph-structured data. This characteristic makes GNNs particularly well suited for analyzing the complex interdependencies often found in malware behavior, which has prompted increasing interest in their application to Android malware detection. For example, Liu et al. [28] introduced a technique for constructing a sensitive FCG by applying graph pruning to preserve security-relevant API contexts while eliminating extraneous nodes. They utilized a word2vec-based embedding strategy for node representation, combined with centrality measures derived from social network analysis, to capture semantic and structural attributes. These were subsequently processed by a graph convolutional network (GCN) to learn graph-level embeddings. Experimental results on the CICMalDroid 2020 dataset demonstrated that this approach achieved an F1 score of up to 98%. In a different line of work, Shen et al. [4] developed a global heterogeneous graph comprising a large number of Android applications and sensitive APIs, designed to capture the complex semantic relationships between APK files and API usage. A multi-layer GCN was employed to learn representation vectors for APKs, which were then fed into a classification model. Their method attained an F1 score of 99.17% on real-world application datasets. Similarly, Zhao et al. [29] proposed a detection framework based on frequent subgraph convolutional neural networks (FSGCNs). They began by constructing Dalvik opcode graphs from Android applications, from which frequent subgraphs were mined and embedded using GCNs to capture salient topological patterns. These embeddings served as inputs to a classifier that distinguishes between benign and malicious apps. The results showed a detection accuracy close to 95%, with an average detection time of under 0.1 s per sample.

A summary of the current research methods for Android malware detection is presented in Table 1.

While current research has predominantly focused on malware detection, the interpretability of detection models is equally critical. Existing interpretability approaches can be broadly categorized into feature-based, rule-based, attention-based, image-based, and graph-based methods [30]. In feature-based approaches, Soi et al. [16] employed SHAP values to analyze both the local and global interpretability of the model. They presented the most influential API call features related to classification outcomes using bar charts and tabular visualizations. XMal [31] combined a multi-layer perceptron (MLP) with an attention mechanism to identify the most important features, which were then mapped to semantic descriptions. Through rule generation and ranking, the system produced natural language explanations that highlighted the core malicious behaviors. In rule-based methods, Yan et al. [32] trained a deep neural network (DNN) and constructed rule trees connecting the input layer to hidden layers (input-hidden trees) and hidden layers to the output layer (hidden-output trees). These were then merged into a comprehensive rule tree that explained the decision-making process of the DNN. Attention-based methods have also been explored. Shamik et al. [33] analyzed gradients across network layers to assess the model’s sensitivity to different parts of the input. Their findings indicated that although the file header often exhibited high gradient values, other segments of the input also significantly influenced the classification result. Furthermore, by performing linear interpolation between samples of different classes and evaluating the model’s responses to the interpolated inputs, they revealed insights into the model’s generalization behavior. In image-based interpretability, Iadarola et al. [34] represented malware samples as images and used deep learning models to predict malware family membership. Grad-CAM was then applied to highlight the image regions most influential to the model’s decision, aiding security analysts in rapidly locating potential malicious code segments. As for graph-based approaches, GAGE [35] leveraged a genetic algorithm to optimize subgraph selection, aiming to identify highly predictive substructures that contribute to malware family classification. This not only improved the interpretability of the model but also enhanced its robustness. In a related study, Liu et al. [28] proposed a visualization-based method that calculates the importance of each node in an FCG to highlight behaviors indicative of malicious activity. Guo et al. [36] proposed a BERT-based attribute-enhanced function embedding method (BBFE) to extract node attributes from call graphs. Subsequently, they designed a hierarchical graph neural network that integrates attention mechanisms and pooling operations. By incorporating function node sampling and structural learning strategies, the framework constructs a comprehensive representation of malicious code behavior.

A summary of the current interpretability methods for Android malware is presented in Table 2.

## 3. GSIDroid

In this section, we provide a detailed introduction to GSIDroid. First, we present an overview of the overall architecture of the detection framework. Then, we delve into each component of the framework, discussing its design principles, implementation details, and roles within the detection process.

### 3.1. Framework

**Figure 1:**
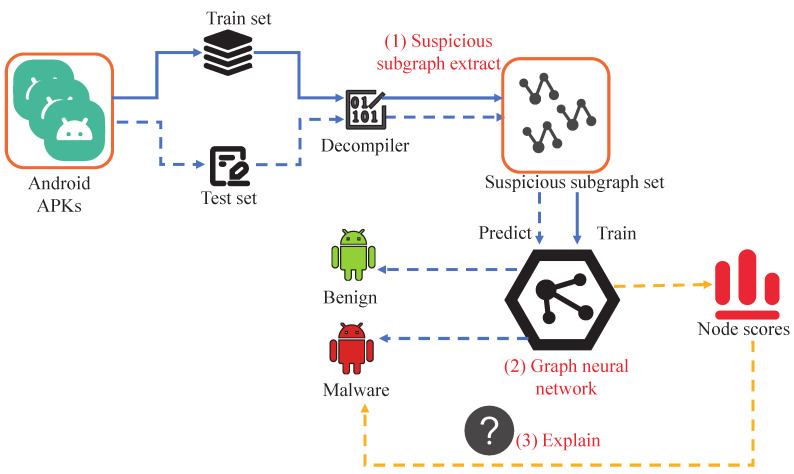
The detection framework of GHIDroid.

Figure 1 illustrates our proposed suspicious subgraph-driven and interpretable Android malware detection system, GSIDroid. The framework consists of three key components: suspicious subgraph extraction, deep- and shallow-semantic–permission fusion architecture, and interpretability analysis.

First, the collected APK files are divided into a training set and a testing set. Then, these APKs are processed using the decompilation tool in conjunction with the suspicious subgraph extraction component. This component focuses on identifying and extracting subgraphs that potentially exhibit malicious behaviors from Android applications. Specifically, it involves constructing the application’s FCG and filtering out subgraphs with potential risks for further analysis. This step forms the foundation of the entire detection process, ensuring that subsequent stages concentrate on the parts most likely to demonstrate malicious behavior. After obtaining the set of suspicious subgraphs, we feed this data into a GNN designed to fuse shallow features with deep semantic information. This network enables effective representation learning of the extracted subgraphs by jointly capturing local details and global patterns, leading to more accurate and efficient subgraph representations. Finally, to enhance the transparency and trustworthiness of the detection results, our framework incorporates a dedicated interpretability analysis module. This module offers both global and local interpretability. Global explanations are achieved by statistically analyzing the permission features associated with the suspicious subgraphs, while local explanations are provided through a node-scoring mechanism that highlights key nodes. This helps security analysts understand why a particular application is flagged as malicious.

### 3.2. Suspicious Subgraph Set Extraction

Android system APIs are a set of programming interfaces provided by the Android operating system that allow applications to control and manage hardware resources [4]. Studies have shown that malicious behaviors are often closely associated with sensitive API calls and the use of high-risk permissions. These features have been proven to be highly effective in malware detection [37,38,39].

Based on these findings, we designed a suspicious subgraph extraction algorithm, as shown in Algorithm 1. Specifically, the algorithm consists of two main parts: suspicious subgraph extraction and node feature assignment.
**Algorithm 1** Suspicious subgraph extraction algorithm    **Input:** APK *A*, Sensitive APIs set *S*, K-hop neighborhood size K=2    **Output:** Suspicious subgraph set *G*    // Suspicious subgraph extraction  1: 
G←ϕ  2: 
$G_{a}=\left(V_{a},E_{a}\right)\leftarrow Decompile\left(A\right)$  3: $V_{s}\leftarrow \left\{v\in V_{a}|v\in S\right\}$ // Obtain the set of function nodes involving sensitive APIs.  4: **for** *v* in $V_{s}$ **do**  5:       $g_{s}=\left(V_{s},E_{s}\right)\leftarrow K-hop\left(G_{a},v,K\right)$ // Extract the subgraph.  6:       $G\leftarrow G\cup \{g_{s}\}$  7: **end for**      // Node feature  8: **for** $g=(V_{g},E_{g})$ in *G* **do**  9:       $F_{g}\leftarrow R^{|V_{g}|\times d}$10:       **for** *v* in $V_{g}$ **do** // Attribute features to each function node.11:              $f_{sem}\leftarrow DexBERT\left(v\right)$12:              $f_{per}\leftarrow CountPermission\left(v\right)$13:              $f\left(v\right)\leftarrow [f_{sem}\left(v\right);f_{per}\left(v\right)]$14:              $F_{g}\left[v\right]\leftarrow f\left(v\right)$15:       **end for**16: **end for**

**Figure 2:**
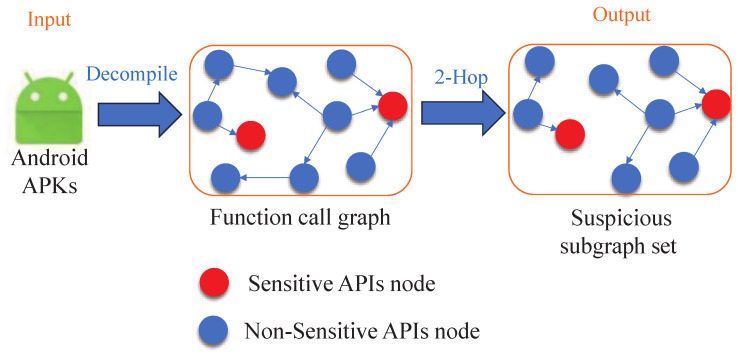
Suspicious subgraph set extraction.

As illustrated in Figure 2, the process of suspicious subgraph extraction begins by decompiling the APK files using the Androguard tool to extract their FCGs. Next, based on the distribution of sensitive API nodes within the call graph, a K-hop algorithm is employed to extract a series of suspicious subgraphs centered around these sensitive API nodes (Algorithm 1, Lines 1–7). By focusing on local structures associated with sensitive APIs, this approach enables precise identification of potentially malicious behavior regions. It avoids redundant computation and unnecessary complexity in analyzing the entire graph, thereby not only improving processing efficiency but also enhancing detection accuracy.

**Figure 3:**
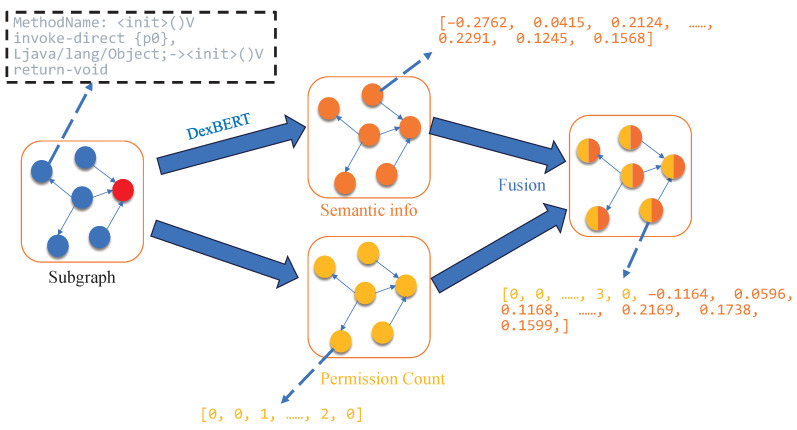
Node feature.

To enrich the feature representation of each node (as illustrated in Figure 3), we take into account that each node corresponds to a specific function. To capture the semantic behavior of these function nodes, we introduce DexBERT [23], a model pre-trained on a large corpus of Smali code. DexBERT provides deep semantic representations that effectively characterize both the internal operations of a function and its interaction patterns with other functions (Algorithm 1, Line 11), enabling fine-grained and accurate semantic descriptions for each node. In addition, the APIs invoked by each node are treated as another important feature source. We count the permissions associated with these APIs to generate permission features (Algorithm 1 Line 12). This means that the model not only considers the functional behavior of each function—captured through DexBERT’s semantic features—but also incorporates its access to system resources, represented by permission features derived from API permission analysis (Algorithm 1 Line 13). By combining both aspects, each node is endowed with a more comprehensive representation, allowing the model to consider richer information dimensions during malware detection.

### 3.3. Deep–Shallow Semantic–Permission Fusion Architecture

**Figure 4:**
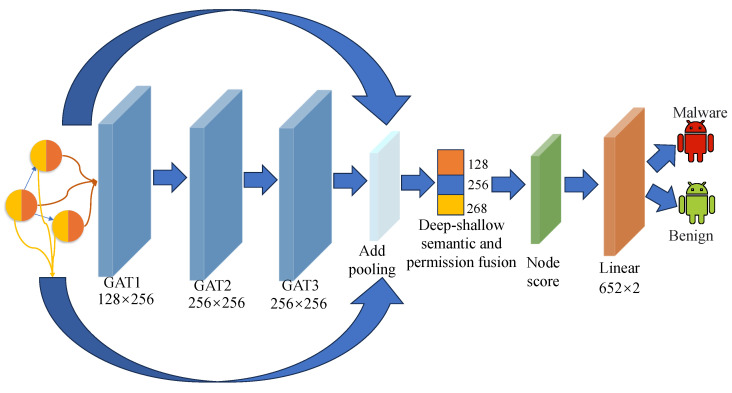
Deep–shallow semantic–permission fusion architecture.

To obtain high-quality graph embeddings, we design a hybrid architecture that integrates both shallow and deep semantic features(as illustrated in Figure 4). Considering the over-smoothing problem commonly encountered in deep GNN, our method first feeds the semantic features of each node into a three-layer graph attention network (GAT). Each GAT layer employs a self-attention mechanism to learn the relationships between nodes, capturing both local and global structural information. After processing through the GAT layers, the deep semantic representations are passed to a node score layer, which computes an importance score for each node. This mechanism highlights critical nodes while suppressing less relevant ones, thereby enhancing the model’s focus on key areas of the graph.

Next, the refined deep semantic features and the original semantic embeddings from DexBERT are separately aggregated using sum pooling, and the resulting representations are concatenated. This ensures that both high-quality semantic embeddings and structural features are preserved.

In addition, we treat the permission features as a form of prior knowledge and incorporate them into the shallow part of the network. This design leverages the critical role of permissions in identifying potential malicious behavior and enhances the model’s understanding of sensitive API usage.

Finally, the shallow-semantic features, deep structural-semantic features, and permission features are fused into a comprehensive feature vector, which is then fed into a fully connected layer for a binary classification task, distinguishing between benign and malicious applications.

#### 3.3.1. Graph Attention Network

The GAT is a deep learning architecture designed for graph-structured data, first proposed by Petar Veličković et al. [40] in 2017. Its core innovation lies in the incorporation of a self-attention mechanism that dynamically computes the importance weights between nodes. This design overcomes the limitations of traditional GCNs, which rely on fixed weight assignments and are tightly coupled with the underlying graph structure.

GAT is capable of handling both inductive and transductive learning tasks, making it highly flexible across various application domains. It has demonstrated strong performance in areas such as molecular modeling, social network analysis, and recommender systems [40,41].

GAT assigns different importance weights to each neighboring node using an attention mechanism, eliminating the need for predefined graph structures or complex matrix operations such as Laplacian matrix decomposition. The workflow of GAT can be divided into the following steps:

1. Linear transformation: Each node’s input features are first linearly transformed to project them into a new feature space:
  (1) $$h_{i}^{\prime }=Wh_{i}$$
  where $h_{i}^{\prime }$ is the feature vector of node *i*, and W is a learnable weight matrix.
2. Attention score computation: Compute the attention score between node *i* and its neighbor node *j*:
  (2) $$e_{ij}=\mathrm{LeakyReLU}(a^{T}[h_{i}^{\prime }\;||\;h_{j}^{\prime }])$$
  where *a* is the learnable attention weight vector.
3. Normalization via Softmax: Normalize the attention scores using the Softmax function to obtain the attention weights:
  (3) $$a_{ij}=\frac{\exp(e_{ij})}{\sum_{k\in N_{i}}\exp\left(e_{ik}\right)}$$
  where $N_{i}$ denotes the set of neighbors of node *i*.
4. Feature aggregation: Aggregate the features of neighboring nodes using the computed attention weights to obtain the updated feature representation for node *i*:
  (4) $$h_{i}^{\prime \prime }=\sigma \left(\sum_{j\in N_{i}}a_{ij}h_{j}^{\prime }\right)$$
  where σ is the activation function.
5. Finally, to improve the stability and expressive power of the model, GAT employs multi-head attention, where multiple independent attention computations are performed in parallel, and their results are averaged:
  (5) $$h_{i}^{\mathrm{final}}=\frac{1}{K}\sum_{k=1}^{K}h_{i}^{\left(k\right)}$$
  where *K* denotes the number of attention heads.

#### 3.3.2. Subgraph Label

Based on the suspicious subgraph extraction algorithm introduced in Section 3.2, we can efficiently obtain a collection of subgraphs from APK files. However, in real-world scenarios, the label information associated with these subgraphs is typically unavailable, which significantly limits their applicability in supervised learning tasks. In other words, it is often infeasible to directly determine whether a given extracted subgraph is benign or malicious. Consequently, the absence of ground-truth labels presents a substantial obstacle to downstream classification, as learning algorithms rely on labeled data to effectively extract discriminative features between benign and malicious behaviors.

To address this challenge, we adopt the loss function defined in MsDroid [17]. This loss function is designed with a specialized optimization objective that enables the model to automatically learn latent malicious patterns in subgraphs without requiring explicit labels. Specifically, for benign samples—whose labels are typically available—the model computes the standard cross-entropy loss (i.e., the first term of the objective function). This is because benign samples often possess reliable annotations and can thus be directly utilized in supervised learning. In this way, the model learns representative features that characterize benign subgraphs.

In contrast, for malicious samples where subgraph-level labels are unavailable, the model instead leverages the predicted probability distribution over subgraphs. It selects those subgraphs deemed most likely to exhibit malicious behavior—namely, those with the highest predicted malicious probability—and computes their associated loss accordingly (i.e., the second term of the objective function).

(6) $$-\left(\left(1-y\right)\frac{1}{\left|G\right|}\sum_{g\in G}\log(1-p_{g})+y\min_{g\in G}\log(p_{g})\right)$$

where *y* denotes the ground-truth label, $p_{g}$ denotes the predicted probability, and *G* denotes the set of subgraphs.

During the testing phase, we introduce a threshold-based decision mechanism to assess the overall maliciousness of an application. For each APK under evaluation, the model performs classification predictions over all extracted subgraphs. If the proportion of subgraphs predicted as malicious exceeds a predefined threshold τ, the application is considered to exhibit pronounced malicious characteristics and is consequently labeled as malware. Otherwise, if the number of malicious subgraphs falls below this threshold, the application is treated as benign, indicating the absence of significant malicious behavior.

(7) $$N_{mal}=\sum_{g\in G}\mathrm{sign}\left(C\left(g\right)\right)$$

(8) $$\mathrm{Label}=\left\{\begin{array}{cc} \mathrm{Malware}, & \mathrm{if}\;N_{mal}>\tau \\ \mathrm{Benign}, & \mathrm{otherwise} \end{array}\right.$$

where *C* denotes the classifier.

### 3.4. Interpretability

In this section, we implement both global and local interpretability methods. For the global interpretability approach, we perform statistical counting of the permission features involved in malicious subgraphs to highlight those permissions that are strongly associated with malicious behaviors.

**Figure 5:**
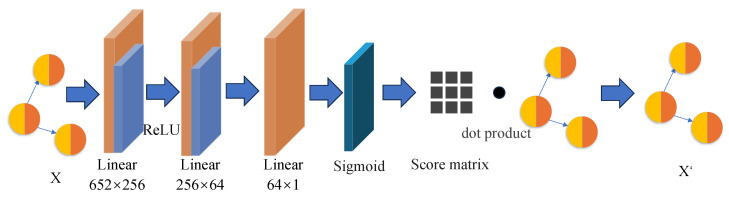
Node score module.

Furthermore, we design and implement a node-level scoring mechanism based on a multi-layer perceptron (MLP) to enable local interpretability, as illustrated in Figure 5. This mechanism is intended to assign a quantitative score to each input node, reflecting its relative importance, and thereby providing support for subsequent interpretability analysis.

Specifically, the implementation of this module proceeds as follows. First, the input data is processed through two fully connected layers, gradually reducing the original 652-dimensional feature space to 64 dimensions. A ReLU activation function is applied after each layer to extract nonlinear representations. Then, a third fully connected layer is used to further compress the feature vector into a single dimension, followed by a Sigmoid activation function to produce a weight score ranging between 0 and 1.

This score directly reflects the importance of each node—the closer the score is to 1, the more important the node is considered to be. A high importance score suggests that the node may be involved in potentially malicious behavior. Thus, our interpretability approach is grounded in node importance. By identifying nodes with higher scores, we are able to highlight those that may harbor latent threats or anomalous activities.

Finally, the generated weight scores are element-wise multiplied with the original node feature matrix to obtain a re-weighted representation. This operation effectively emphasizes the contribution of key nodes while suppressing the influence of irrelevant or low-importance nodes.

## 4. Experiment Settings

To ensure diversity and authenticity in our experimental samples, we collected APK files from multiple sources, including AndroZoo [42], CICMalDroid2020 [43,44], and VirusShare. These datasets comprise a wide range of APK files, encompassing various malicious behavior patterns and benign application characteristics.

In total, we collected 14,520 samples, including 7625 malicious samples obtained from CICMalDroid2020 and VirusShare, covering multiple malware families, and 6895 benign samples sourced from AndroZoo and CICMalDroid2020. To eliminate the influence of duplicate applications on the experimental results, we performed signature and package name checks to ensure no redundancies existed in the dataset. The detailed dataset composition is presented in Table 3.

To evaluate the performance of our model, we partitioned the dataset into training and testing sets in an 8:2 ratio. Specifically, the training set contains 11,616 samples (9220 benign and 7300 malicious), while the testing set includes 2904 samples (1675 benign and 1229 malicious).

We adopt standard metrics to evaluate the performance of our models in the experiments. As shown in Table 4, the core evaluation components include true positive (TP), false negative (FN), true negative (TN), and false positive (FP), as well as additional indicators such as time and memory consumption.

A true positive refers to a sample that is actually positive and correctly predicted as positive. A false negative indicates a sample that is actually positive but incorrectly predicted as negative. A true negative refers to a sample that is actually negative and correctly predicted as negative. A false positive indicates a sample that is actually negative but incorrectly predicted as positive. These statistical outcomes are summarized via a confusion matrix, as illustrated in Table 5.

Accuracy measures the proportion of correctly classified samples among all samples. Precision evaluates the proportion of true positives among all samples predicted as positive. Recall measures the proportion of true positives among all actual positives. The F1 score is the harmonic mean of precision and recall.

In addition, we introduce a series of metrics related to time and memory consumption. Training time refers to the amount of time required to train a classifier using the training dataset, which includes the time for CPU data loading, GPU training, and other associated processes. Testing time denotes the time needed for the trained classifier to predict the input samples. Training memory consumption refers to the amount of memory utilized by the system to store and process the training dataset, model parameters, and intermediate computations during the training phase. Testing memory consumption indicates the amount of memory required by the trained classifier to perform predictions on input samples, including the storage of input data and model weights.

The hardware and software configurations used in our experiments are summarized in Table 6 and Table 7, respectively.

The hyperparameters for the model are presented in Table 8. The optimizer used is Adam with a learning rate of 0.001, training for 200 epochs. The number of attention heads is set to 2, and the detection threshold τ is set to 0.1.

## 5. Experiment Results

### 5.1. Overall Performance

To verify the effectiveness of GHIDroid, we compared it with several classic and state-of-the-art approaches, including DAPASA [45], MamaDroid [37], $S^{3}$ Feature [46], and MsDroid [17]. These methods were selected due to their similarity to our work, primarily in utilizing API permission information or employing graph structures for malicious behavior detection.

DAPASA captures the most suspicious behavioral patterns within applications by constructing sensitive subgraphs (SSGs). MamaDroid extracts API call sequences from applications and abstracts them into representations at class, package, or family levels, subsequently modeling these sequences using Markov chains to capture application behavior features. $S^{3}$ Feature achieves feature extraction by constructing a sensitive function call graph (SFCG) and mining SSG within it. MsDroid focuses on local code fragments around sensitive APIs, representing each fragment as a graph encoding opcode and permission information, and employs GNN for classification.

During the experiments, we trained and evaluated these methods using their open-source implementations available on GitHub, which we applied to our collected dataset.

The experimental results presented in Table 9 illustrate performance differences across various evaluation metrics among the compared models. Although DAPASA achieves a high precision of 99.4%, it performs inadequately in other metrics, indicating a conservative approach that struggles to effectively identify a large number of malicious samples. In comparison, MamaDroid demonstrates a more balanced performance, highlighting the effectiveness of API call sequences in capturing malicious behavior. Despite adopting a similar methodology to DAPASA, the $S^{3}$ feature delivers superior performance. Instead of merely extracting five features from sensitive subgraphs as DAPASA does, the $S^{3}$ feature constructs feature representations by integrating SSG and neighbor-sensitive graphs (NSGs), providing richer information sources for distinguishing between malicious and benign applications. MsDroid leverages GNN from deep learning; however, its overall performance is below expectations, possibly due to limitations from insufficient sample size and architectural constraints.

**Figure 6:**
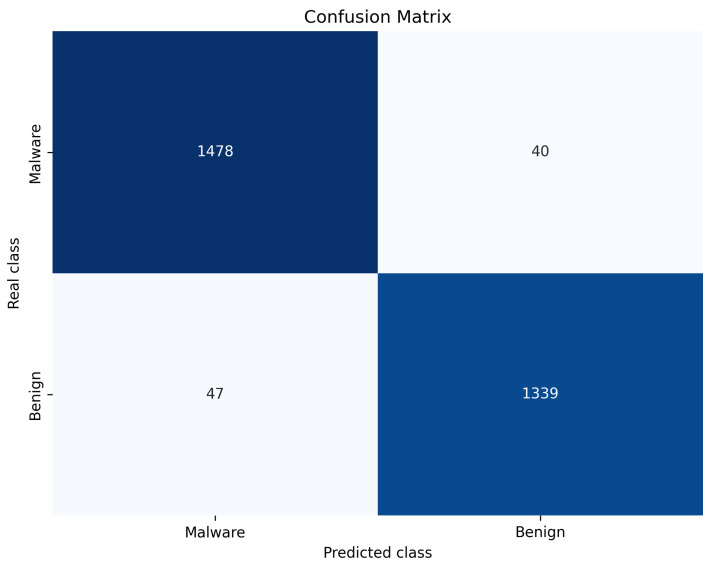
Confusion matrix of GSIDroid.

Our proposed GHIDroid model achieves the highest performance, with a recall of 96.92%, an accuracy of 97%, and an F1 score of 97.14%. Figure 6 presents the corresponding confusion matrix, confirming the advantage of integrating both shallow and deep semantic features along with permission information in improving detection accuracy.

Furthermore, from the perspective of time and memory consumption, DAPASA, MamaDroid, and the $S^{3}$ feature, which use traditional machine learning models, have significantly lower computational overhead. Their training times are under 33 s with memory usage below 53 MB, and testing memory is minimal. In contrast, MsDroid and our proposed GHIDroid, both based on GNNs, consume far more resources. MsDroid uses 265.12 s and 59.8 GB for training, while GHIDroid requires 279.58 s and 62.1 GB. Because MsDroid employs a simpler three-layer GCN structure, its efficiency in terms of time and memory is slightly higher, but its accuracy still remains lower than that of GHIDroid.

### 5.2. Ablation Experiment

To verify the influence of deep semantic features, shallow-semantic features, and permission information on classification performance, we designed and conducted a series of ablation experiments. By systematically removing or retaining specific types of input features, we evaluated their individual contributions to the final classification results. The experimental design was as follows: we first established a base model containing only deep semantic features, and then separately incorporated shallow-semantic and permission features. The experimental results are summarized in Table 10.

As indicated by the data presented in Table 10, compared to the base model, incorporating either shallow-semantic (SS) information or permission characteristics (PC) improves the model’s performance. Specifically, when only shallow-semantic features were added (base + SS), the model’s accuracy increased from 95.7% to 96.63%, an improvement of 0.93 percentage points, and the F1 score improved from 95.86% to 96.76%, a gain of 0.9 percentage points. This demonstrates the significant role of shallow-semantic features in enhancing the model’s overall classification capability.

Further analysis reveals that introducing only permission features (base + PC) slightly elevated the model’s accuracy to 95.87%, an increase of 0.17 percentage points over the base model. Similarly, the F1 score rose to 96.02%, an improvement of 0.16 percentage points. These results suggest that permission features also contribute effectively to model performance. Although the magnitude of improvement was relatively small, it clearly highlights their distinctive value in malware detection.

Most notably, combining both shallow-semantic information and permission features (base + SS + PC) led to the greatest performance improvement. In particular, accuracy reached 97%, representing a 1.3-percentage-point increase over the base model, and the F1 score achieved 97.14%, which is 1.28 percentage points higher. This outcome not only underscores the importance of each individual feature type but also illustrates their synergistic effect when used together, resulting in substantial enhancements in both accuracy and comprehensive evaluation metrics.

## 6. Interpretability

In this section, we explain the model’s decision-making process using the method introduced in Section 3.4 and illustrate its practical effectiveness through a concrete APK example.

**Figure 7:**
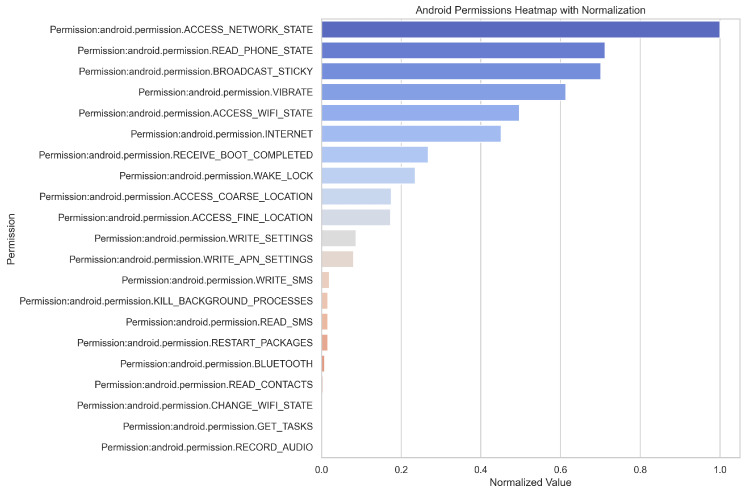
Global interpretation.

To facilitate global interpretation, we first analyzed permission usage statistics, with the results illustrated in Figure 7. The frequent occurrence of permissions such as ACCESS_NETWORK_STATE, INTERNET, and ACCESS_WIFI_STATE suggests that malicious applications often require stable network connections to communicate with remote servers—either to exfiltrate stolen data or to receive command-and-control instructions.

The presence of READ_PHONE_STATE indicates a tendency of malware to harvest device identifiers (e.g., IMEI, carrier information), while the use of RECEIVE_BOOT_COMPLETED and WAKE_LOCK reflects attempts to ensure persistent background activity by enabling automatic startup upon device boot. This behavior prolongs the malware’s residence time and enhances concealment.

Furthermore, the use of ACCESS_FINE_LOCATION and ACCESS_COARSE_LOCATION reveals a capacity for fine-grained location tracking. Permissions such as RECORD_AUDIO, READ_SMS, and WRITE_SMS imply potential for intercepting voice and text communications, raising significant privacy concerns. Finally, the presence of WRITE_SETTINGS, WRITE_APN_SETTINGS, and KILL_BACKGROUND_PROCESSES suggests that the malware possesses the capability to modify system configurations and disrupt other applications, which may be leveraged to escalate privileges, maintain control, or destabilize the system.

Furthermore, it can be observed from Figure 7 that the proportion of the VIBRATE permission is relatively high. However, since this permission is widely used in applications and has low sensitivity—such as for call notifications, alert vibrations, or haptic feedback in games—it frequently appears but is generally not regarded as an indicator of malicious behavior.

**Figure 8:**
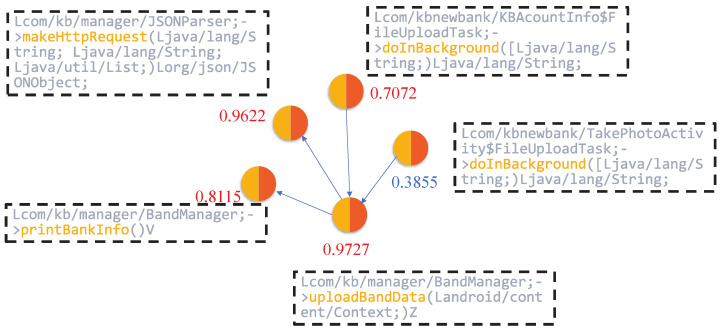
Suspicious subgraph extracted from the apk (2426ff25f8814d77fb25cb45ed41f3bb77ce1f3c51c88d5495d923528050d591).

Subsequently, we employed the node-scoring module to generate local interpretations by quantifying the importance of input features. This process assigns a contribution weight to each node, indicating its relative impact on the model’s prediction. As illustrated in Figure 8, nodes such as JSONParser.makeHttpRequest, KBAcountInfo & FileUploadTask.doInBackground, BandManager.uploadBandData, and BandManager.printBankInfo received high importance scores of 0.9622, 0.7072, 0.9727, and 0.9115, respectively.

These nodes are closely associated with operations involving sensitive permissions or critical API calls, including access to device storage, network communication, and retrieval of user privacy data. The elevated scores suggest that the model regards these functions as highly influential in identifying malicious behavior, thus validating the effectiveness of the interpretability framework in highlighting security-relevant components.

**Figure 9:**
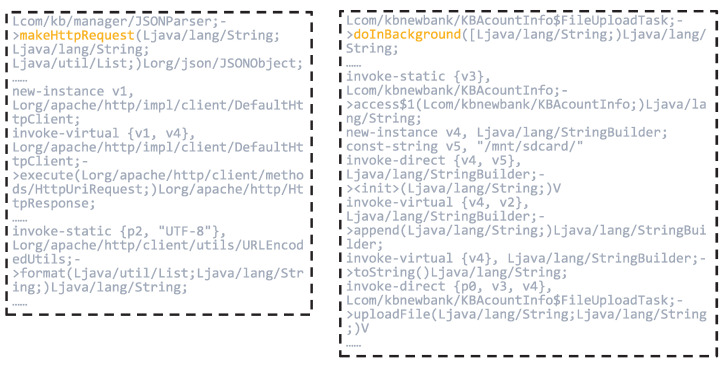
Partial Code of the JSONParser.makeHttpRequest and KBAcountInfo & FileUploadTask.doInBackground Functions.

**Figure 10:**
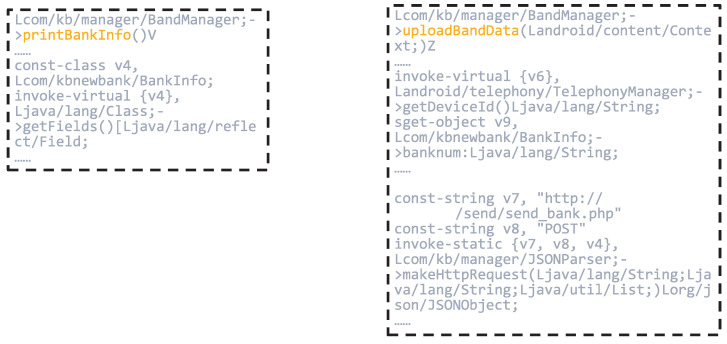
Partial Code of the BandManager.uploadBandData and BandManager.printBankInfo Functions.

Figure 9 and Figure 10 provide partial code listings for the functions JSONParser.makeHttpRequest, KBAcountInfo & FileUploadTask.doInBackground, BandManager.uploadBandData, and BandManager.printBankInfo.

Focusing on JSONParser.makeHttpRequest, this method illustrates the use of DefaultHttpClient to initiate HTTP requests, with parameters encoded using URLEncodedUtils.format. It takes a URL, an HTTP method (e.g., POST), and a list of parameters as input, then transmits the request to the specified server. Further inspection reveals that this method is used to send data to the endpoint http://*.*.*.*/send/send_bank.php (The domain information has been anonymized), suggesting its potential role in transmitting sensitive information to external servers.

The KBAcountInfo & FileUploadTask.doInBackground function defines an asynchronous task responsible for constructing a file path and invoking the uploadFile method. The use of external storage directories (e.g., /mnt/sdcard/) indicates that the application is likely attempting to access and upload files stored locally on the device, which raises potential concerns regarding data leakage.

The BandManager.printBankInfo function leverages Java reflection to enumerate all fields within the BankInfo class. While this mechanism is commonly used for debugging or development purposes, its presence in production code may introduce security risks. Specifically, it can be exploited to bypass conventional access controls and expose sensitive information, such as banking credentials or account details.

Moreover, the BandManager.uploadBandData function retrieves the device’s IMEI number and aggregates it with the banknum field, subsequently transmitting this data via the previously discussed makeHttpRequest method. This behavior suggests that the application not only collects device-specific identifiers but also potentially exfiltrates sensitive financial information to external servers, thereby posing a significant threat to user privacy.

In conclusion, the combined behavior of these functions reveals a concerning pattern, that is, the application is likely collecting sensitive personal data—such as banking account information and unique device identifiers—without the user’s knowledge or consent. This data is then transmitted to unidentified third-party servers via insecure communication channels. Such activities represent a severe breach of user privacy principles and demonstrate a clear malicious intent, potentially constituting unauthorized data harvesting and misuse. These findings highlight the importance of incorporating interpretability into malware detection frameworks, as they enable the identification of covert and privacy-invasive behaviors within seemingly benign applications.

## 7. Conclusions and Future Work

In this paper, we presented GSIDroid, an innovative and interpretable Android malware detection framework driven by suspicious subgraphs. The proposed system focuses on extracting suspicious subgraphs centered around sensitive API calls and integrates both deep and shallow-semantic features along with permission information into a graph neural network (GNN) model. By combining global explanations based on permission statistics with local interpretations derived from node-level scoring, GSIDroid not only achieves effective malware detection but also identifies key nodes that significantly influence classification outcomes, thereby greatly enhancing the interpretability of the model.

This interpretability is crucial for security analysts seeking to understand and investigate malicious behavior patterns in depth. Experimental results demonstrate that by integrating semantic and permission-based features, GSIDroid achieves superior detection performance, outperforming several state-of-the-art baselines. These findings suggest that our approach not only improves detection accuracy but also deepens the scope and granularity of malware behavior analysis.

While GSIDroid demonstrates strong performance and interpretability, it is not without limitations. One notable drawback lies in the computational complexity associated with graph construction and suspicious subgraph extraction, which can lead to increased time overhead. To mitigate this, a promising direction for future research is the development of more efficient and precise subgraph extraction algorithms, with the goal of reducing computational costs and improving overall processing efficiency.

In addition, the semantic feature extraction process relies on the DexBERT model, whose large-scale results in a highly time-consuming embedding process. A potential direction for future work is to apply model distillation techniques to DexBERT, aiming to produce a lightweight variant that enables faster inference while maintaining sufficient accuracy.

In conclusion, while GSIDroid has made significant advancements in the field of Android malware detection, there remains room for improvement. We anticipate that addressing the current bottlenecks in the framework will further enhance its efficiency and practicality. Looking ahead, we aim to continue advancing this line of research to develop more effective solutions against the increasingly sophisticated threats posed by modern malware.

## Figures and Tables

**Table 1:** Summary of representative Android malware detection methods.

Method	Year	Model	Feature	Data Source	Performance
SegDroid [28]	2024	GCN	Pruned FCG	CICMalDroid 2020	F1 score: 98%
GHG-Droid [4]	2024	GCN	Heterogeneous Graph, API Semantics	Multiple datasets	F1 score: 99.17%
Zhao et al. [29]	2025	FSGCN	Opcode Graph, Frequent Subgraphs	Drebin, MobileSandbox, Google Play	F1 score: 94.4%
DexRay [20]	2021	1D CNN	Bytecode Image	AndroZoo	F1 score: 96%
Zhu et al. [18]	2023	CNN	Bytecode Image	VirusShare, Google Play	F1 score: 95.9%
Tang et al. [19]	2024	CNN	Mixed Image Features	Drebin, CICMalDroid 2020	Accuracy: 98.67%
Basak et al. [21]	2024	DRIN	Bytecode Image	Malshare, VirusShare, VirusTotal	F1 score: 99.48%
DexBERT [23]	2023	BERT + MLP	Pre-trained BERT on Opcode	MYST (Malicious Code Localization)	F1 score: 99.81%
LTA-Checker [22]	2024	LSTM + TCN + Multi-head Attention	Opcode Sequence	CIC-InvesAndMal2019, RmvDroid, MalRadar, AndroZoo	F1 score: 98.69%
MAPAS [27]	2022	CNN	API Call Graph	VirusShare, Google Play	Accuracy: 93.2%
Zhu et al. [25]	2023	CNN	Permission, API Calls, Hardware Features	VirusShare	F1 score: 96.07%
Dong et al. [26]	2024	CNN, DNN	Permission, API Call Graph	Drebin, Google Play	F1 score: 93.55%
Yang et al. [24]	2024	SVM	API Semantics	AndroZoo, VirusTotal, Google Play	Accuracy: 96.7%

FSGCN: frequent subgraph GCN; DRIN: dynamic residual involution network.

**Table 2:** Summary of interpretability methods for Android malware detection.

Method	Year	Type	Technique
XMal [31]	2021	Feature-based + Attention	Attention
Soi et al. [16]	2024	Feature-based	SHAP Values
Yan et al. [32]	2021	Rule-based	Rule Tree
Bose et al. [33]	2020	Attention-based	Gradient
Iadarola et al. [34]	2021	Image-based	Grad-CAM
GAGE [35]	2024	Graph-based	Genetic Algorithm
Liu et al. [28]	2024	Graph-based	Node Importance
Guo et al. [36]	2025	Graph-based + Attention	Attention

**Table 3:** The number of apps used in our experiments.

Source	Type	Counts
AndroZoo	Benign	3940
CICMalDroid2020	Benign	2955
VirusShare	Malware	2506
CICMalDroid2020	Malware	5119

**Table 4:** Evaluation metrics.

Indicator	Abbreviation	Meaning
True Positive	TP	Malicious samples are correctly classified as malicious.
True Negative	TN	Benign samples are correctly classified as benign.
False Positive	FP	Benign samples are incorrectly classified as malicious.
False Negative	FN	Malicious samples are incorrectly classified as benign.
Accuracy	Acc	(TP+TN)/(TP+TN+FP+FN)
Precision	P	TP/(TP+FP)
Recall	R	TP/(TP+FN)
F1 score	F1 score	2∗P∗R/(P+R)
Training time	Tra_time	-
Testing time	Tes_time	-
Training memory consumption	Tra_mc	-
Testing memory consumption	Tes_mc	-

**Table 5:** Confusion matrix.

	Predicted Class	
Real Class	Malware	Benign
Malware	TP	FN
Benign	FP	TN

**Table 6:** Hardware configuration.

Item	Configuration
CPU	Intel Xeon W-2275
RAM	128 GB
GPU	NVIDIA RTX A4000

**Table 7:** Software version.

Item	Configuration
Operating system	Ubuntu 20.04
Python	3.8.18
PyTorch	2.0.0
Androguard	3.3.5
scikit-learn	1.3.1

**Table 8:** Hyperparameter settings.

Item	Configuration
Optimizer	Adam
Learning rate	0.001
Epoch	200
attention heads *K*	2
τ	0.1

**Table 9:** The overall performance and comparisons.

Methods	Year	Precision	Recall	Accuracy	F1 Score	Tra_time	Tes_time	Tra_mc	tes_mc
DAPASA	2017	99.4	77.98	88.72	87.4	3.23 s	5.2 µs	24.2 MB	0.01 MB
MamaDroid	2019	94.78	85.7	90.01	90.01	8.35 s	15.32 µs	32.67 MB	0.01 MB
$S^{3}$ Feature	2022	95.09	95.28	94.94	95.19	32.75 s	23.32 µs	52.25 MB	2 MB
MsDroid	2023	81.03	90.95	84.37	85.73	265.12 s	17.36 s	59.8 GB	502 MB
GHIDroid	2025	97.36	96.92	97	97.14	279.58 s	24.86 s	62.1 GB	531 MB

**Table 10:** Comparison among GHIDroid variants.

Variants	Precision	Recall	Accuracy	F1 Score
base	96.85	94.89	95.7	95.86
base + SS	97.53	96	96.63	96.76
base + PC	97.05	95.02	95.87	96.02
base + SS + PC	97.36	96.92	97	97.14

SS: Shallow-semantic information. PC: permission characteristics.

## Data Availability

Malware data used in this study are publicly available from their respective owners. References have been provided throughout the manuscript to enable the reader to reach out to the original authors.
